# Dietary total antioxidant capacity and risk of prediabetes and diabetes mellitus: a systematic review and dose-response meta-analysis of 170,919 participants

**DOI:** 10.3389/fnut.2025.1541734

**Published:** 2025-02-25

**Authors:** Ruomeng Li, Long Shu, Qin Zhu, Dan Lu

**Affiliations:** ^1^Department of Digestion, Zhejiang Hospital, Hangzhou, China; ^2^Department of Nutrition, Zhejiang Hospital, Hangzhou, China

**Keywords:** dietary total antioxidant capacity, prediabetes, diabetes mellitus, systematic review, dose-response meta-analysis

## Abstract

**Background:**

Observational studies have assessed the association between total antioxidant capacity of the diet and risk of diabetes mellitus. However, results from these studies were not entirely consistent. In the current systematic review and dose-response meta-analysis, we aimed to determine the association between dietary total antioxidant capacity (TAC) and the risk of prediabetes and diabetes mellitus.

**Methods:**

A systematic literature search of authentic electronic resources including PubMed/Medline, Embase, Scopus, ISI Web of Science and China National Knowledge Infrastructure (CNKI) was carried out to find the relevant articles published up to November 2024. Random-effects or fixed-effects models were used to aggregate the relative risks (RRs) and their 95% confidence intervals (CIs) where appropriate. Heterogeneity across the studies were determined using the Cochran’s *Q* test and *I*-square (*I*^2^) statistics.

**Results:**

A total of 10 observational studies (five cohort, three case-control and two cross-sectional studies) were included in our meta-analysis. The pooled results indicated that higher dietary TAC was significantly associated with lower risk of prediabetes (RR = 0.58; 95% CI: 0.34–0.97; *p* = 0.039) and diabetes mellitus (RR = 0.71; 95% CI: 0.58–0.87, *p* = 0.001). In addition, dose-response analysis showed a linear trend association between dietary TAC and risk of diabetes mellitus (RR = 0.928; 95% CI: 0.842–1.023, *p*_dose-response_ = 0.131, *p*_nonlinearity_ = 0.078). Subgroup analyses showed the significant inverse association between dietary TAC and diabetes mellitus in mean age <50 and sample size <5,000 (RR = 0.26, 95% CI: 0.16–0.41, *p* < 0.001), and there was no evidence of heterogeneity (*p* = 0.939; *I*^2^ = 0.0%). Meanwhile, there was also an inverse association between dietary TAC and diabetes mellitus in Western countries (RR = 0.79; 95% CI: 0.68–0.92, *p* = 0.003), with less evidence of heterogeneity (*p* = 0.226; *I*^2^ = 36.7%).

**Conclusion:**

Overall, higher dietary TAC was inversely associated with the risk of prediabetes and diabetes mellitus. Further well-designed prospective studies or randomized controlled trials are needed to validate the present findings.

**Systematic Review Register:**

(PROSPERO), CRD42024611235.

## Introduction

Globally, diabetes mellitus, as one of the most common and fastest growing chronic non-communicable diseases, has become a serious public health problem (1). According to the latest estimates from International Diabetes Federation (IDF) in 2021, the global prevalence of diabetes mellitus will reach to be 12.2%, projecting to affect 783.2 million adults (aged 20–79 years) by 2045 (2). Given the growing incidence and healthcare burden of diabetes mellitus, urgent public health preventive measures are of particular importance. As is known to all, diabetes mellitus is a chronic, multifactorial disorder that may be associated with various risk factors, including genetic predisposition and environmental factors, e.g., physical inactivity, cigarette smoking, and dietary factors (3, 4).

Over the past few decades, increasing evidence suggests that dietary factors, particularly those rich in antioxidants, may play a crucial role in the pathogenesis of diabetes mellitus (5). For example, a previous systematic review and meta-analysis revealed that greater intake of green leafy vegetables was associated with a 14% reduced risk of type 2 diabetes mellitus (6). The researchers speculated that the antioxidant vitamins in vegetables might play a key role in this protective effect (7). Antioxidants are known to inhibit the activity of free radicals and reduce oxidative stress (7), an important risk factor in the pathogenesis of diabetes mellitus (8). The majority of previous observational studies have commonly focused on the impact of individual antioxidants intake on diabetes mellitus risk (9–12). For example, Golmohamadi et al. (11), in a prospective cohort study, found that dietary vitamin E significantly decreased the risk of diabetes mellitus. However, research on individual antioxidants may not fully capture the cumulative or synergistic effects of various antioxidants in the overall diet (13). In view of this, dietary total antioxidant capacity (TAC) has been designed as a direct measurement tool for assessing the diet’s total antioxidant potential, considering the possible interactions between antioxidant nutrients in food (14).

Currently, dietary TAC is receiving extensive attention and interest in epidemiological research (15). Growing evidence shows that higher dietary TAC intake has been inversely related to various negative health outcomes, including cardiovascular diseases, pancreatic cancer and mortality (16–18). Notably, a more recent systematic review and meta-analysis shows that higher intake of dietary TAC is associated with a reduced risk of stroke (14). However, epidemiological studies on the correlation between dietary TAC and diabetes mellitus risk are quite limited. Up to now, only eight previous studies have reported the association between dietary TAC and risk of diabetes mellitus (7, 19–25), but their findings were not entirely consistent. Although several studies have shown the protective effect of dietary TAC against diabetes mellitus (7, 22), other studies found no significant association (19, 20, 23). Furthermore, to our knowledge, no previous systematic review and dose-response meta-analysis has been carried out to comprehensively evaluate the links between dietary TAC and prediabetes and diabetes mellitus risk. Therefore, to identify the exact associations between dietary TAC and prediabetes and diabetes mellitus risk, we performed this systematic review and dose-response meta-analysis to summarize the findings from observational studies published up to November 2024.

## Methods

### Protocol and registration

This study was performed in accordance with the Meta-analysis of Observational Studies in Epidemiology (MOOSE) guidelines (26), and the protocol has been registered on November 17, 2024 in the International Prospective Register of Systematic reviews (PROSPERO) database with registration number CRD42024611235.

### Search strategy

A systematic search using PubMed/MEDLINE, ISI Web of Science, Embase, Scopus and CNKI databases was performed to find the studies published that have evaluated the associations between dietary TAC and prediabetes and diabetes mellitus up to November 2024. The search strategy included the predefined keywords: “dietary total antioxidant capacity,” “dietary TAC,” “dietary antioxidant capacity,” “non enzymatic antioxidant capacity,” “dietary antioxidant index,” “antioxidant capacity of diet” and “diabetes mellitus,” “diabetes,” “insulin resistance,” “hyperglycemia,” “prediabetes.” The detailed search strategy of each database is available in Supplementary Table 1.

Moreover, hand-searching from reference lists of all relevant articles, previous reviews and meta-analyses was performed to identify relevant studies. At the same time, unpublished studies or grey literature were not eligible in this meta-analysis.

### Study selection

Two authors (RL and LS) independently examined and cross-checked the titles and abstracts of all published articles retrieved in the initial search, and removed duplicates and irrelevant articles. The full-text versions of these articles were then reviewed according to the inclusion and exclusion criteria for this study. In order to be included in our analyses, studies must meet all of the eligibility criteria: (1) observational studies, including cohort, case-control or cross-sectional studies; (2) those adult participants aged ≥18 years; (3) the main exposure of interest was dietary TAC; (4) the outcome of interest was prediabetes and/or diabetes mellitus; (5) provided the multivariable adjusted effect estimates in the form of HRs, RRs, or ORs with 95% CIs (or sufficient data to calculate them); (6) if the retrieved studies lacked sufficient detail, the corresponding author of eligible study were contacted by email. Additionally, exclusion criteria was as follows: (1) non-observational studies, such as reviews, case reports, letters and editorials; (2) did not provide the HRs, RRs or ORs with corresponding 95% CIs; (3): the exposure of interest was single antioxidant, such as vitamin C, vitamin E; (4) irrelevant articles. When the results of a study in men and women are reported separately, we treated each analysis as a separate study. Any discrepancy was resolved by discussion or in consultation with the corresponding author (DL). The study population, exposure, comparison, outcome, and study design (PECOS) information is illustrated in Table 1.

**Table 1:** The PECOS criteria used for this systematic review and dose-response meta-analysis.

Population	Adults
Exposure	Dietary total antioxidant capacity
Comparison	Highest category vs. lowest category of exposure
Outcomes	Diabetes mellitus and prediabetes
Study design	Observational studies with the design of cohort, case-control or cross-sectional

PECOS, participant, intervention (exposure), comparison, outcome, and study design.

### Data extraction

Two authors (RL and LS) performed data extraction independently, including first author’s last name, publication year, study design, study region, sample size, mean age/age range, follow-up time in cohort studies, methods for dietary assessment, numbers of participants and prediabetes/diabetes mellitus cases, confounding variables adjusted for the multivariate analyses, and reported risk estimates with their corresponding 95% CIs of prediabetes/diabetes mellitus across categories of dietary TAC.

### Quality assessment

Two authors (QZ and LS) used the Newcastle–Ottawa Scale (NOS) to evaluate the quality of each selected article in this study. The NOS scale was designed for non-randomized studies in meta-analyses, composing of eight items in three domains: selection (4 points), comparability (2 points), and ascertaining of the outcome (3 points), with a maximum score of nine (27). Studies with NOS scores ≥7 points were recognized as high quality (28). Any discrepancies between two authors were resolved by the corresponding author to reach a consensus.

### Definition of dietary TAC

Dietary TAC is designed as a direct measurement tool to evaluate total antioxidant potential of the whole diet using different chemical methods, such as the ferric reducing antioxidant potential (FRAP), the oxygen radical absorbance capacity (ORAC), the trolox equivalent antioxidant capacity (TEAC), the total radical-trapping antioxidant parameter (TRAP), and vitamin C equivalent antioxidant capacity (VCEAC) (14).

### Data synthesis and statistical analyses

In main analyses, we used RR and 95% CI as the primary effect size. Additionally, we considered that HR was approximately equal to RR (29). OR was converted into RR using the following formula: RR = OR/[(1 − *P*_0_) + (*P*_0_ × OR)], where *P*_0_ shows the incidence of diabetes mellitus in the non-exposed group (30). We carried out a pairwise meta-analysis that pooled the RRs and 95% CIs of the highest and lowest categories of dietary TAC with prediabetes and diabetes mellitus risk. Between-study heterogeneity was assessed by Cochran’s *Q* test and quantified by the *I*^2^ statistics. A *p*-value of *Q*-test >0.10 or *I*^2^ < 50% showed an absence of heterogeneity among the included studies, and fixed-effects model was used to pool RRs. Due to expected heterogeneity between the included studies, RRs were calculated using the random-effects model (DerSimonian and Laird methods) (31). When results showed the significant heterogeneity, sensitivity and subgroup analyses were performed to identify the potential sources of heterogeneity. In our analyses, subgroup analyses were performed to explore potential effects attributable to variables such as study design (cohort or case-control/cross-sectional studies), sex (men or women), study region (Western or Asian countries), study quality (≥7 or <7), outcome (type 2 diabetes or gestational diabetes), mean age (≥50 years or <50 years), sample size (<5,000 or ≥5,000), and methods for dietary assessment (FFQ or others). A sensitivity analysis was performed to confirm whether the combined RRs had a robust or sensitive effect on a single study or a group of studies. Publication bias was assessed through visual inspection of the funnel plots and quantified by both Begg’s and Egger’s regression asymmetry tests (32). If there was publication bias, the trim and fill method was used to recalculate the combined RRs (33). At the same time, we performed a dose-response meta-analysis to estimate the trend from the correlated log RRs across the categories of dietary TAC scores. A two-stage GLST model based on generalized least squares was used to examine the linear or non-linear dose-response association between dietary TAC and diabetes mellitus risk (34). In this study, we used dietary TAC model and restricted cubic splines with three knots at fixed percentiles (10, 50, and 90%) distributions. Analyses were conducted using STATA/SE, version 14.0 (StataCorp, College Station, TX, United States). All statistical tests were two-sided, and *p*-values <0.05 were considered to be statistically significant unless otherwise specified.

## Results

### Search results

**Figure 1:**
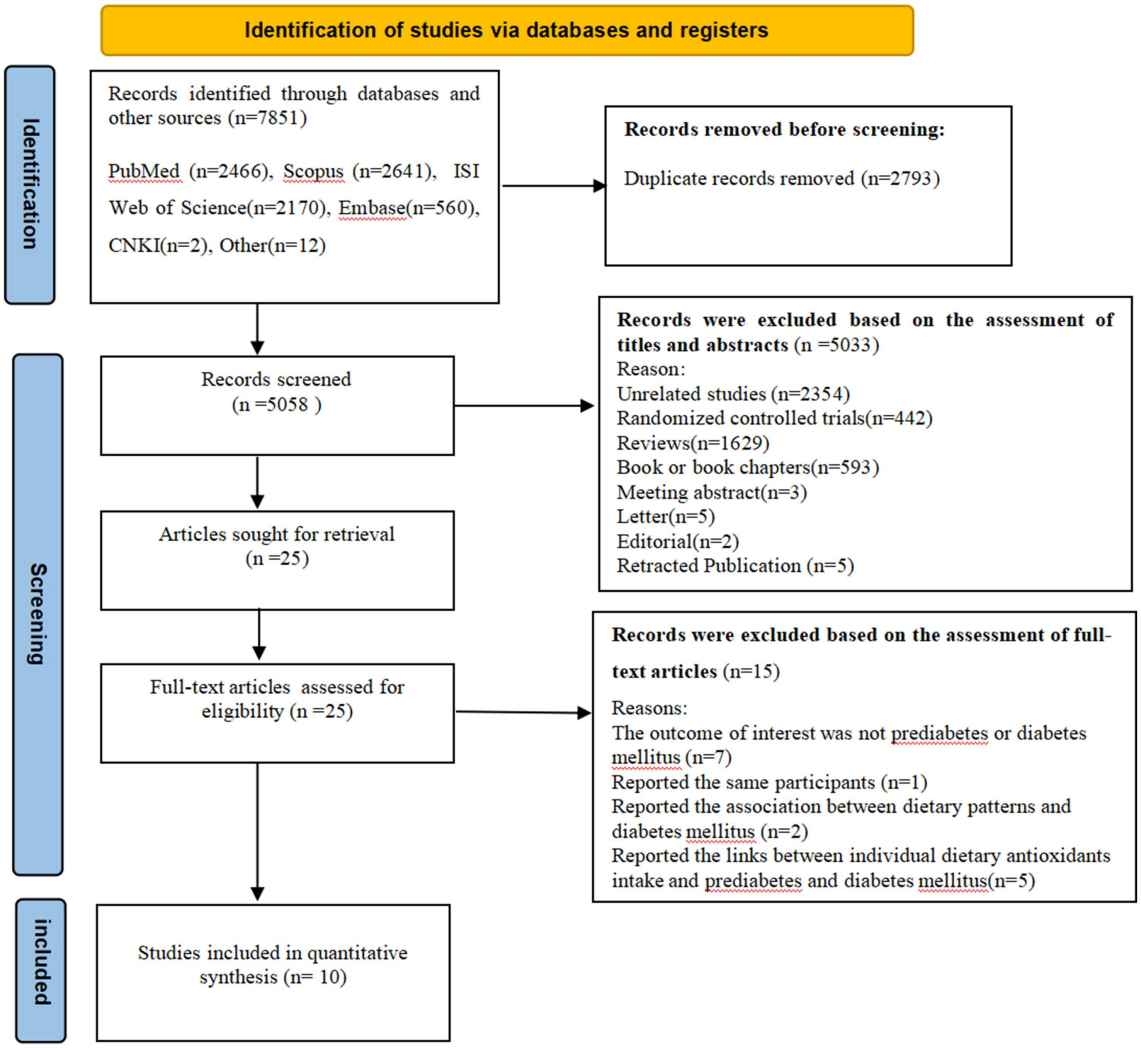
Flow chart of the process of the study selection.

The flow chart of literature search process is shown in Figure 1. The systematic search and reference list screening yielded 7,851 articles. After omitting 2,793 duplicates, 5,058 articles were remained for further assessment. Subsequently, 5,033 articles were excluded based on the assessment of titles and abstracts of retrieved articles. Twenty-five full-text articles were assessed for eligibility. Of the remaining 25 articles, 15 were excluded because of the following reasons: outcomes of interest were not prediabetes or diabetes mellitus (*n* = 7), reported the same participants (*n* = 1), reported the association between dietary patterns and diabetes mellitus (*n* = 2), and reported the links between individual dietary antioxidants intake and diabetes mellitus (*n* = 5). Ultimately, 10 articles were included in the analyses (7, 19–25, 35, 36). The PECOS for the present meta-analysis is shown in Table 1.

### Study characteristics

Characteristics of eligible studies were presented in Table 2. A total of 10 articles was included, with sample size of included studies ranging from 147 to 64,660. Of these included studies, five were cohort studies (7, 21–24), three were case-control studies (25, 35, 36) and two were cross-sectional studies (19, 20). With regards to the origin of the studies, four studies were carried out in Iran (24, 25, 36, 37), one in China (20), one in Korea (21), one in France (22), one in Japan (23), one in Netherlands (7), and one in Poland (19). All the included studies were published between 2018 and 2024. Seven studies included both men and women (7, 19–21, 23, 35, 36), and remaining three studies only included women (22, 24, 25). The follow-up duration for the cohort studies ranged from 1 to 15 years. The age of participants across studies ranged from ages 18 to above. Seven of included studies used FFQ to collect dietary data (7, 20, 21, 23, 24, 35, 36), two studies used 24-h dietary recall (19, 25), and one study used dietary questionnaire (22). To calculate the quantitative value of dietary TAC, six studies used FRAP assay (7, 19, 20, 22, 24, 35), one study used ORAC assay (36), one study used VCEAC (21) one study used FRAP, TRAP and TEAC assays (25), and one study used FRAP, TRAP and ORAC assays (23). Based on the NOS, from all included studies, nine studies were high quality (7, 19–24, 35, 36), and the remaining one study was medium quality (25). The quality assessment of included studies bases on NOS criteria is shown in Table 3.

**Table 2:** Characteristics of the included studies on the association between dietary total antioxidant capacity and prediabetes and diabetes mellitus risk.

Author, publication year	Study region	Study design	Total number of participants	Age	Dietary assessment method	Adjustment or matched for in the analyses	Outcomes
van der Schaft et al., 2019 (7)	Netherlands	Cohort	5,796 (532 cases)	≥45 years	FFQ	Age, sex, Rotterdam study cohort, body mass index, hypertension, dyslipidaemia, highest level of education attained, physical activity, smoking status, degree of adherence to dietary guidelines, total daily energy intake and daily alcohol intake	HR = 0.84, 95% CI: 0.75–0.95; men HR = 0.84, 95% CI: 0.71–1.00; women HR = 0.83, 95% CI: 0.70–0.99
Cyuńczyk et al., 2022 (19)	Poland	Cross-sectional	413 (40 cases)	35–65 years	24 h dietary recalls	Age, sex, family history of diabetes, educational level, smoking status, physical activity, dyslipidemia, hypertension, BMI, waist circumference, alcohol consumption, daily energy intake	Highest quartile 4 vs. lowest quartile 1 of TAC (OR = 0.237, 95% CI: 0.037–1.516)
Li et al., 2024 (20)	China	Cross-sectional	12,467 (1,238 cases)	≥18 years	FFQ	Age, sex, smoking status, alcohol consumption, physical activity, BMI, WC, TG, HDL-C, hypertension and health supplement intake	Highest quartile 4 vs. lowest quartile 1 of TAC (OR = 0.96, 95% CI: 0.80–1.17)
Tan et al., 2022 (21)	Korea	Cohort	20,594 (692 cases)	40–79 years	FFQ	Age, body mass index (BMI), educational level, physical activity, drinking status, smoking status and total energy intake	Highest quintile vs. lowest quintile of TAC: men HR = 0.73, 95% CI: 0.50–1.06; women HR = 0.58, 95% CI: 0.40–0.83; per SD increment of TAC: men HR = 0.85, 95% CI: 0.75–0.96; women HR = 0.81, 95% CI: 0.71–0.92
Mancini et al., 2018 (22)	France	Cohort	64,223 (1,751 cases)	52 years	Dietary questionnaire	Smoking status (never smoker vs. ever smoker), physical activity (MET-h/week), education level (less or more than 14 years of education), hypertension, hypercholesterolaemia (self reported blood cholesterol >6.2 mmol/L or use of cholesterol-lowering drugs: yes vs. no), family history of diabetes (yes vs. no), energy intake (kJ/day), alcohol intake (g ethanol/day), adherence score for the healthy dietary pattern and adherence score for the western dietary pattern	Highest quintile vs. lowest quintile of TAC: HR = 0.73, 95% CI: 0.60–0.89
Kashino et al., 2019 (23)	Japan	Cohort	64,660 (1,191 cases)	44–76 years	FFQ	Age, sex, public health center area, smoking status, total physical activity, history of hypertension, family history of diabetes, coffee consumption, energy intake, use of supplements and BMI	Highest quintile vs. lowest quintile of TAC (OR = 1.04, 95% CI: 0.88–1.23)
Heshmati et al., 2024 (24)	Iran	Cohort	1,856 (369 cases)	18–45 years	FFQ	Body mass index (kg/m^2^), occupation, age, hypertension, diabetes, education, and working rotating shift	Highest quartile 4 vs. lowest quartile 1 of TAC (RR = 0.29, 95% CI: 0.12–0.68); per SD increment of TAC: (RR = 0.66, 95% CI: 0.48–0.90)
Daneshzad et al., 2020 (25)	Iran	Case-control	463 (200 cases)	22–44 years	24 h dietary recalls	Age, energy intake, Socioeconomic status, number of offspring, dietary fiber intake, carbohydrate, protein intake, BMI, supplementation, physical activity, and fat intake	Highest tertile 3 vs. lowest tertile 1 of TAC (OR = 0.15, 95% CI: 0.08–0.29)
Rahmani et al., 2021 (35)	Iran	Case-control	147 (49 cases)	18–90 years	FFQ	Age, gender, BMI, marital status, income, occupation, education, physical activity, dietary supplementation, family history of diabetes, and total calorie intake	Highest tertile 3 vs. lowest tertile 1 of TAC (OR = 0.09, 95% CI: 0.02–0.53)
Sotoudeh et al., 2018 (36)	Iran	Case-control	300 (150 cases)	30–65 years	FFQ	BMI, physical activity, education, dietary intake of fiber, fat, energy. and coffee	Highest quartile 4 vs. lowest quartile 1 of TAC (OR = 0.18, 95% CI: 0.07–0.49)

BMI, body mass index; CI, confidence interval; FFQ, food frequency questionnaire; HDL-C, high-density lipoprotein-cholesterol; HR, hazard ratio; MET, metabolic equivalents; OR, odds ratio; TAC, total antioxidant capacity; TG, triglyceride; WC, waist circumference.

**Table 3:** Dietary total antioxidant capacity and risk of diabetes mellitus: assessment of study quality.

Studies	Selection				Comparability		Outcome			Score
	1	2	3	4	5A	5B	6	7	8	
Cohort										
van der Schaft et al., 2019 (7)	*	*	*	*	*	*	*	*	*	9
Tan et al., 2022 (21)	*	*	*	*	*		*	*	*	8
Mancini et al., 2018 (22)	*	*	*	*	*	*	*	*	*	9
Kashino et al., 2019 (23)	*	*	*	*	*		*	*	*	9
Heshmati et al., 2024 (24)	*	*	*	*	*		*	*	*	8
Case-control/cross-sectional										
Cyuńczyk et al., 2022 (19)	*	*	*		*		*	*		6
Li et al., 2024 (20)	*	*	*		*		*	*	*	7
Daneshzad et al., 2020 (25)	*	*	*		*		*	*	*	7
Rahmani et al., 2021 (35)	*	*	*		*		*	*	*	7
Sotoudeh et al., 2018 (36)	*	*	*		*		*	*	*	7

*For case-control studies, 1 indicates cases independently validated; 2, cases are representative of population; 3, community controls; 4, controls have no history of diabetes mellitus; 5A, study controls for the most important factor; 5B, study controls for additional factor(s), e.g., cigarette smoking body mass index, total energy intake; 6, ascertainment of exposure by blinded interview or record; 7, same method of ascertainment used for cases and controls; and 8, non response rate the same for cases and controls. For cohort studies, 1 indicates exposed cohort truly representative; 2, non exposed cohort drawn from the same community; 3, ascertainment of exposure by secure record (e.g., surgical records) or structured interview; 4, outcome of interest was not present at start of study; 5A, study controls for the most important factor; 5B, study controls for additional factor(s); 6, assessment of outcome is based on independent blind assessment or record linkage; 7, follow-up long enough (≥5 years) for outcomes to occur; and 8, adequacy of follow up of cohorts (all participants complete follow up or >90% participants complete follow up).

### Dietary TAC and prediabetes risk

**Figure 2:**
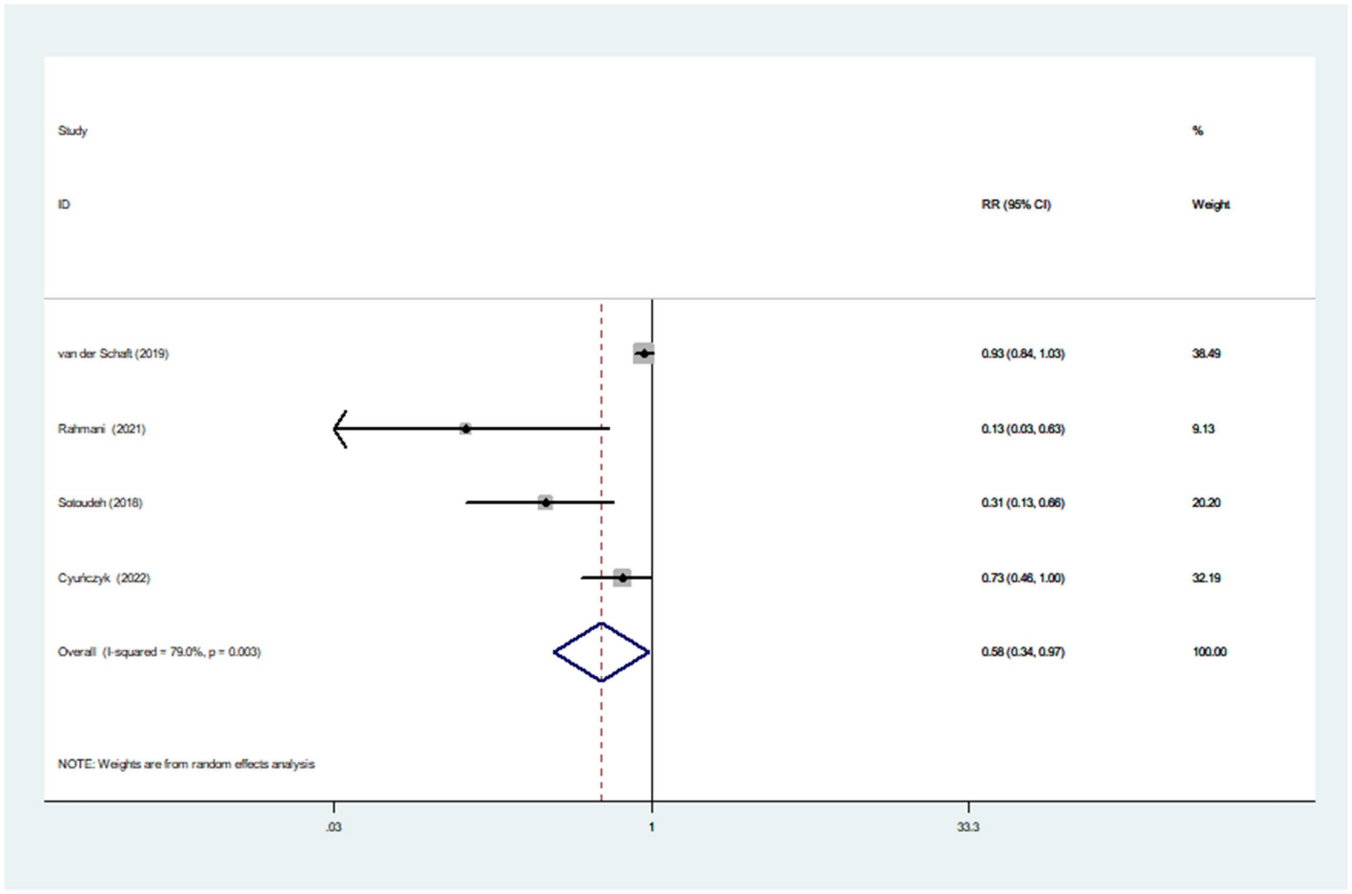
Forest plot of the association between dietary TAC intake and risk of prediabetes.

Four studies involving 5,817 participants and 1,195 cases, were included to evaluate the association between dietary TAC and prediabetes risk. Figure 2 showed that higher dietary TAC intake was associated with the reduced risk of prediabetes (RR = 0.58; 95% CI: 0.34–0.97; *p* = 0.039), with significant heterogeneity between included studies (*I*^2^ = 79.0%, *p* = 0.003). Thus, the effect size was evaluated using the random-effects models. Due to the limited data, we were unable to conduct subgroup analyses to explore potential sources of heterogeneity across studies.

### Dietary TAC and diabetes mellitus risk

**Figure 3:**
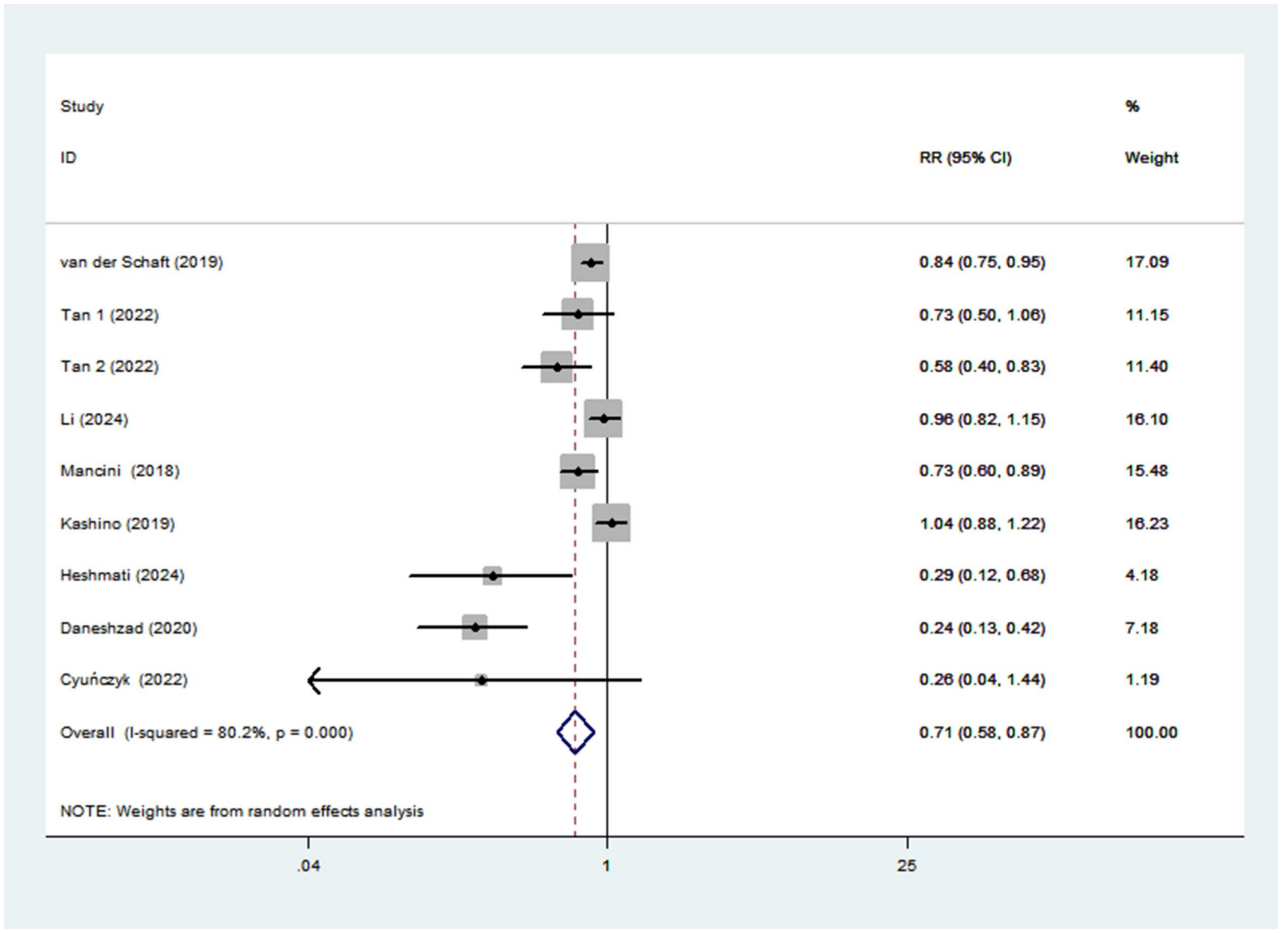
Forest plot of the association between dietary TAC intake and risk of diabetes mellitus.

**Figure 4:**
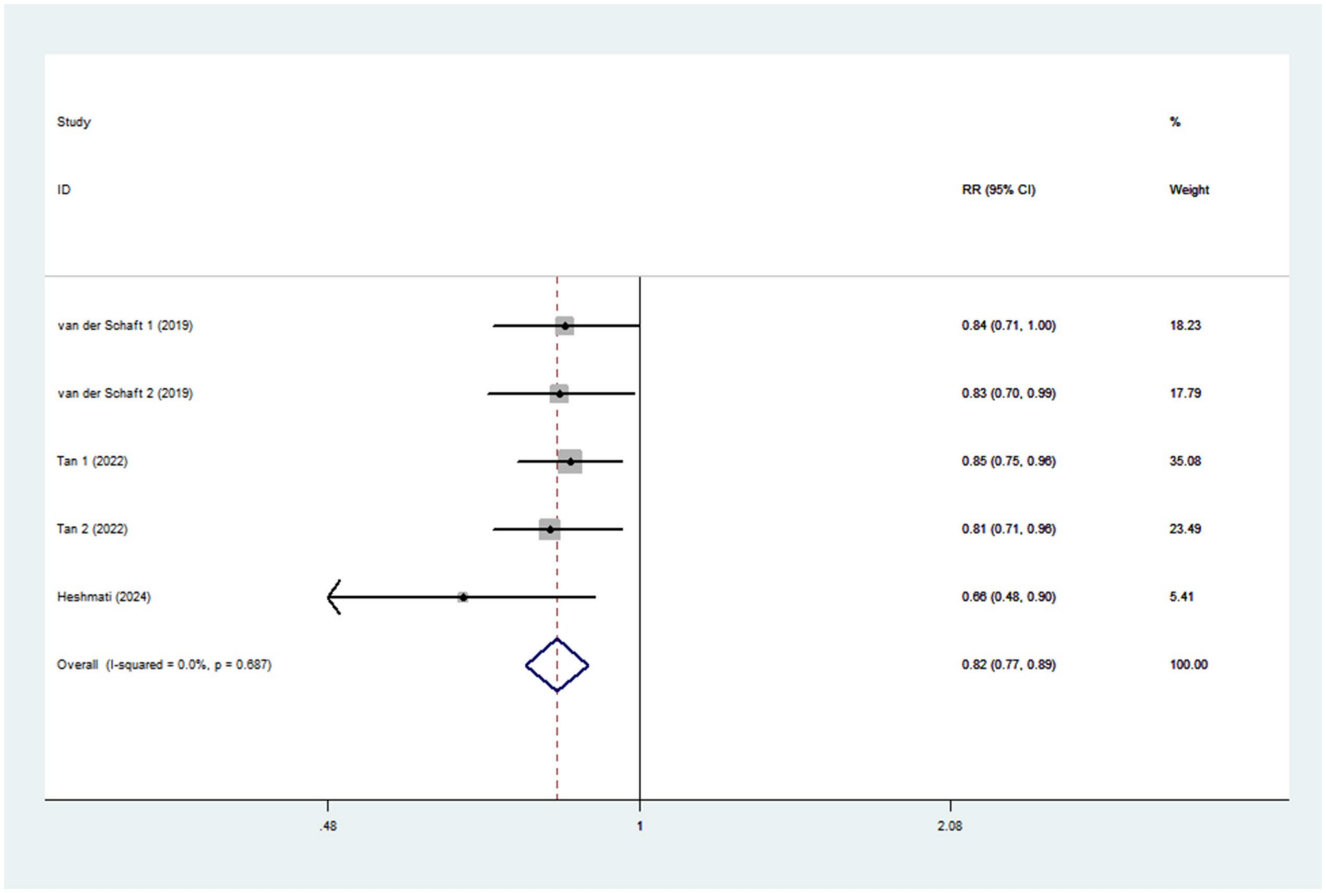
Forest plot of the association between each 1 SD increment in dietary TAC intake and risk of diabetes mellitus.

Eight studies involving 170,472 participants and 6,013 cases, were included to evaluate the association between dietary TAC and diabetes mellitus risk. Combining nine effect sizes from eight studies, Figure 3 shows the evidence of a 29% lower risk of diabetes mellitus in the highest compared with the lowest categories of dietary TAC scores (RR = 0.71; 95% CI: 0.58–0.87, *p* = 0.001). The significant heterogeneity was found in the included studies (*I*^2^ = 80.2%; *p* < 0.001), thus a random-effects model was applied to pool RRs. In addition, Figure 4 showed that each SD increment in dietary TAC intake was associated with a 18% lower risk of diabetes mellitus (RR = 0.82; 95% CI: 0.77–0.89, *p* < 0.001; *I*^2^ = 0.0%; *p* = 0.687).

### Dose-response analysis

**Figure 5:**
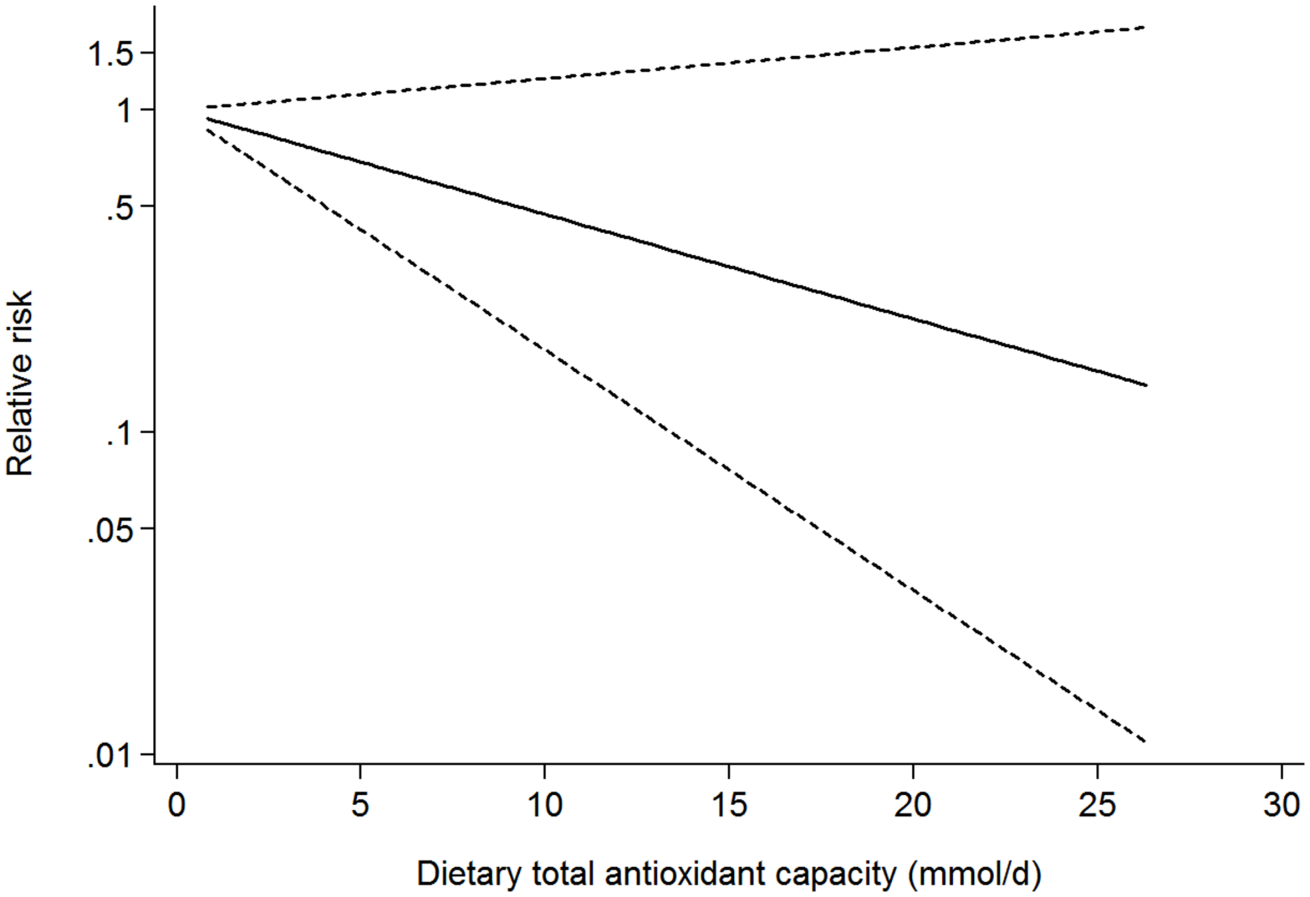
Dose-response analysis for the association between dietary TAC intake and risk of diabetes mellitus.

Seven studies (four cohort, two case-control, and one cross-sectional studies) were included in the dose-response analysis for the association between dietary TAC and risk of diabetes mellitus (Figure 5). The dose-response analysis indicated a linear trend association between dietary TAC and risk of diabetes mellitus (*p*_nonlinearity_ = 0.078). However, this dose-response association was not statistically significant (RR = 0.928; 95% CI: 0.842–1.023, *p*_dose-response_ = 0.131), possibly because the number of studies is small.

### Subgroup analyses

To further identify the potential sources of heterogeneity across included studies, we conducted subgroup analyses basing on study design, sex, study region, study quality, outcome, mean age, sample size, and methods for dietary assessment (Table 4). The results showed a significant inverse association between dietary TAC and diabetes mellitus in studies with mean age <50 and sample size <5,000 (RR = 0.26, 95% CI: 0.16–0.41, *p* < 0.001), and there was no evidence of heterogeneity (*p* = 0.939; *I*^2^ = 0.0%). In addition, there was also an inverse association between dietary TAC intake and diabetes mellitus in Western countries (RR = 0.79; 95% CI: 0.68–0.92, *p* = 0.003), with less evidence of heterogeneity (*p* = 0.226; *I*^2^ = 36.7%).

**Table 4:** Subgroup analyses of diabetes mellitus for the highest versus lowest categories of dietary TAC intake.

Dietary total antioxidant capacity	Subgroup	No. of studies	RR (95% CI)	*p*-values	Heterogeneity		
					*p*-values for within groups	*I*^2^ (%)	*p-*values for between groups
Study design	Cohort	5	0.76 (0.63–0.93)	0.006	0.002	73.4	0.807
	Case-control/cross-sectional	3	0.43 (0.13–1.39)	0.160	<0.001	90.7	
Sex	Men	3	0.87 (0.75–1.01)	0.078	0.288	19.6	0.157
	Women	6	0.63 (0.46–0.88)	0.006	<0.001	85.2	
Study region	Western countries	3	0.79 (0.68–0.92)	0.003	0.226	32.7	0.265
	Asian countries	5	0.64 (0.45–0.89)	0.008	<0.001	86.2	
Study quality	≥7	6	0.66 (0.52–0.85)	0.001	<0.001	83.3	0.113
	<7	2	0.69 (0.22–2.10)	0.509	0.155	50.6	
Outcome	Type 2 diabetes	6	0.83 (0.72–0.96)	0.010	0.011	63.7	<0.001
	Gestational diabetes	2	0.25 (0.16–0.41)	<0.001	0.723	0.0	
Mean age	≥50 years	5	0.84 (0.73–0.96)	0.013	0.011	66.3	<0.001
	<50 years	3	0.26 (0.16–0.41)	<0.001	0.939	0.0	
Sample size	≥5,000	5	0.84 (0.73–0.96)	0.013	0.011	66.3	<0.001
	<5,000	3	0.26 (0.16–0.41)	<0.001	0.939	0.0	
Methods for dietary assessment	FFQ	5	0.82 (0.68–0.98)	0.029	0.003	72.0	0.003
	24 h dietary records/dietary questionnaire	3	0.40 (0.15–1.02)	0.056	0.001	85.1	

CI, confidence interval; dietary TAC, dietary total antioxidant capacity; FFQ, food frequency questionnaire; RR, relative risk.

### Publication bias

As shown in Supplementary Figure 1, inspection of funnel plots revealed little evidence of asymmetry. Begg’s test showed no evidence of publication bias had no statistical significance (highest compared with lowest categories of dietary TAC: *p* = 0.076). However, Egger’s test for publication bias had statistical significance (*p* = 0.021). Thus, we applied the trim and fill analysis to re-estimate the pooled RRs (Supplementary Figure 2). After performing trim and fill analysis, the results showed that no study was added to the funnel plot and no change in the overall RRs was found (RR = 0.71; 95% CI: 0.58–0.87, *p* < 0.01).

### Sensitivity analysis

In sensitivity analysis (Supplementary Figure 3), the results showed that the association between dietary TAC and diabetes mellitus risk was robust and not affected by any single study or a couple of studies.

## Discussion

To the best of our knowledge, this is the first systematic review and dose-response meta-analysis to comprehensively evaluate the association between dietary TAC and risk of prediabetes and diabetes mellitus. In this study, our results indicated that higher dietary TAC intake was significantly associated with lower risk of prediabetes and diabetes mellitus. Moreover, the dose-response analysis also indicated a linear trend association between dietary TAC and risk of diabetes mellitus. Similarly, sensitivity analysis did not show the significant impact of any single study on the pooled results. Our findings validate the results of previous observational studies and underscore the clinical importance of higher dietary TAC intake in the prevention of prediabetes and diabetes mellitus.

In the past few decades, the global epidemic of diabetes mellitus has continued to increase (37). According to the latest estimates from IDF in 2021, the global prevalence of diabetes mellitus will reach to be 12.2%, projecting to affect 783.2 million adults by 2045 (2). Considering the enormous burden to public healthcare systems, identifying risk factors and implementing prevention are the main strategies for controlling diabetes mellitus. It is well established that among modifiable factors for diabetes mellitus, dietary factors have attracted more attention. However, many observational studies have highlighted the important role of single dietary antioxidants (e.g., polyphenols, vitamin C and minerals) in the prevention of diabetes mellitus (9–11, 38, 39), and examining the antioxidant capacity of the overall diet remains limited. To date, few epidemiological studies have investigated dietary TAC in relation to prediabetes and diabetes mellitus (7, 19–25), but the results are inconsistent. For example, in a French E3N-European Prospective Investigation into Cancer and Nutrition (EPIC) cohort, the total antioxidant capacity of the diet was inversely associated with type 2 diabetes in middle-aged women (22). Similarly, van der Schaft et al. (7) also observed that higher total dietary antioxidant capacity was associated with a reduced risk of type 2 diabetes among the total population. In contrast, Kashino et al. (23), in the Japan Public Health Center-based Prospective Study, failed to observe any significant association between dietary NEAC and type 2 diabetes. It is worth noting that in this meta-analysis, we found that higher dietary TAC intake was inversely associated with the risk of prediabetes and diabetes mellitus. The variations in these published studies may be ascribed to the following several reasons. First, the difference in dietary habits may contribute the inconsistent results. In all included studies, four studies were carried out in Iran, where dietary habits are different to the Western countries, such as France. Second, methods for measuring the dietary TAC are also different. For example, six of included studies used FRAP assay to measure the dietary TAC (7, 19, 20, 22, 24, 35), one used ORAC assay (36), and one used VCEAC (21). Thus, different methods might affect the true antioxidant capacity of overall diet. Third, the major contributors to dietary TAC were inconsistent across countries and regions. For instance, in Bialystok Polish Longitudinal University Study (PLUS) population, Cyuńczyk et al. (19), reported that the principal food sources of dietary TAC were coffee infusion, fruits and juices, tea infusion, nuts and seeds and vegetables without potatoes. However, in middle-aged French women, Mancini et al. (22) reported that the main contributors to dietary TAC were fruit, vegetables, alcoholic beverages and hot beverages without coffee. Notably, coffee has been reported to be the main contributor of dietary TAC in many countries (7, 22). Fourth, discrepancies in dietary assessment methods may contribute to the differing results. Daneshzad et al. (25) and Cyuńczyk et al. (19), used 24-h dietary recalls to obtain dietary intake information, Mancini et al. (22), used dietary questionnaire, and the remaining seven studies used FFQs (7, 20, 21, 23, 24, 35, 36). Taken together, variabilities in the dietary habits across countries, methods for measuring dietary TAC, dietary assessment methods, and types of foods may contribute to the different results.

Although evidence on the correlation between dietary TAC and risk of diabetes mellitus remains inconsistent, several potential mechanisms could be put forward to explain the protective effect. Firstly, previous studies have documented that dietary antioxidants can prevent oxidative stress (40), an important contributing factor in the pathogenesis of prediabetes and type 2 diabetes mellitus (41). Secondly, available evidence indicates that antioxidants, e.g., vitamin C, E and carotenoids, rich in fruits and vegetables are associated with a decreased risk of hypertension, which is implicated in the development of type 2 diabetes (42). Simultaneously, a recent updated systematic review and meta-analysis also showed that higher intake of dietary TAC was associated with reduced fasting blood sugar (43). Thirdly, higher dietary TAC intake has been reported to be related to the lower plasma concentration of high-sensitivity C-reactive protein (44). The evidence shows that increased inflammation can impair the secretion of insulin, leading to insulin resistance (45), a well-established risk factor for type 2 diabetes mellitus (46). Fourthly, as mentioned above, coffee is a major contributor of dietary TAC. Prior studies have confirmed the beneficial effect of coffee intake on the prevention of type 2 diabetes (47). Taken together, the above mechanisms may explain the beneficial effect of high dietary TAC intake against diabetes mellitus.

Our meta-analysis showed the protective associations between high dietary TAC intake and the risk of prediabetes and diabetes mellitus, but significant heterogeneity was also observed for prediabetes (*I*^2^ = 79.0%; *p* = 0.003) and diabetes mellitus (*I*^2^ = 80.2%; *p* < 0.001), respectively. For this reason, we carried out subgroup analyses to explore the possible sources of heterogeneity. In our analyses, subgroup analyses were performed based on study design, sex, study region, study quality, outcome, mean age, sample size, and methods for dietary assessment. The results showed that significant heterogeneity could be explained by the differences in study region, sample size, mean age, and outcome. When subgroup analyses were performed in a subgroup of mean age <50 and sample size <5,000, heterogeneity of this study decreased from 80.2 to 0.0%. Indeed, several plausible explanations have been put forward. First, RRs, HRs or ORs were from highest category vs. lowest category. However, different studies measuring the dietary TAC used different methods, such as FRAP assay or ORAC assay, and divided dietary TAC range into different intervals. These might result in significant heterogeneity. Second, all included studies were performed in Iran (24, 25, 36, 37), China (20), Korea (21), France (22), Japan (23), Netherlands (7), and Poland (19), respectively. These countries often have their own unique eating habits, which may lead to significant heterogeneity. Third, different confounding variables adjusted in the eligible studies may contribute to significant heterogeneity. Fourth, five of included studies were case-control and cross-sectional studies, which were more prone to recall and selection biases. We therefore could not determine the causality of observed relationship. Thus, the significant inverse associations observed in subgroups with small sample sizes (<5,000) should be taken with caution. Additionally, information on dietary intake were collected through FFQs and 24 h dietary recall, which might also lead to recall and selection biases. Finally, significant heterogeneity remained in the other subgroup analyses, indicating that there may be other unknown confounding factors.

### Strengths and limitations

This meta-analysis has several strengths and limitations. First, this is the first systematic review and dose-response meta-analysis that has assessed the association between dietary TAC and risk of prediabetes and diabetes mellitus. Our findings add the available evidence for the favorable effect of higher dietary TAC intake on diabetes mellitus, and underscore the importance of higher dietary TAC intake in the prevention of diabetes mellitus. Second, all eligible articles have been strictly screened basing on our inclusion and exclusion criteria. Third, incident cases of prediabetes and diabetes mellitus were ascertained through medical records review, reducing possibility of misdiagnosis. Fourth, subgroup and sensitivity analyses further improved the accuracy of our findings. Finally, we also conducted a dose-response analysis to strengthen the relationship between dietary TAC and risk of diabetes mellitus. Nonetheless, several possible limitations should be acknowledged when interpreting the findings. First, due to the observational nature of included studies, no causal relationship can be drawn from the results. Meanwhile, half of included studies were case-control and cross-sectional studies. Thus, we could not fully rule out recall and selection biases. Further prospective cohort studies or randomized controlled trials are necessary to validate the results of this study. Second, in this study, dietary data was collected by using the self-completed FFQ or 24 h dietary records, which might lead to the under-or over-estimations of dietary TAC. As such, measurement errors were inevitable. Third, even though adjusting for some established potential confounders, residual confounding from undetected or unknown factors could not be excluded. Moreover, differences in adjustment for potential confounders (e.g., dietary patterns, physical activity, or socioeconomic status) across studies might affect the reliability of our findings. Fourth, significant heterogeneity was observed in our analyses. Although subgroup and sensitivity analyses were carried out to find the possible sources of heterogeneity, we were unable to fully explain the sources of inter-study heterogeneity. Finally, more than half of included studies were performed in Asian and Middle Eastern countries, limiting the generalizability of our findings to the broader population of Western countries.

## Conclusion

In conclusion, this study showed that higher dietary TAC intake was inversely associated with the risk of prediabetes and diabetes mellitus. Our findings confirmed the existing evidence on the beneficial effects of dietary TAC on prediabetes and diabetes mellitus, and underscored the importance of higher dietary TAC intake in the prevention of diabetes mellitus. Moreover, our findings also support public health recommendations to encourage the consumption of dietary TAC. Nevertheless, further well-designed prospective studies or randomized controlled trials are needed to confirm these findings.

## Data Availability

The original contributions presented in the study are included in the article/Supplementary material, further inquiries can be directed to the corresponding author.

## Glossary

ABTS: 2,2′-azino-bis (3-ethylbenzthiazoline) 6-sulfonic acid
CI: Confidence interval
CNKI: China National Knowledge Infrastructure
CVD: Cardiovascular disease
EPIC: European Prospective Investigation into Cancer and Nutrition
FFQ: Food frequency questionnaire
FRAP: Ferric reducing antioxidant potential
GBD: Global Burden of Disease Study
HR: Hazard ratio
IDF: International Diabetes Federation
NEAC: Non-enzymatic antioxidant capacity
NOS: Newcastle–Ottawa Quality Scale
OR: Odds ratio
ORAC: Oxygen radical absorbance capacity
PRISMA: Preferred Reporting Items for Systematic Reviews and Meta-Analyses
RR: Relative risk
TAC: Total antioxidant capacity
TEAC: Trolox equivalent antioxidant capacity
TRAP: Total radical-trapping antioxidant parameter
VCEAC: Vitamin C equivalent antioxidant capacity
