# Supplementary figures and images for: Dietary total antioxidant capacity and risk of prediabetes and diabetes mellitus: a systematic review and dose-response meta-analysis of 170,919 participants
Source: Front Nutr. 2025 Feb 25;12:1541734. doi: 10.3389/fnut.2025.1541734 (PMC11893433; doi:10.3389/fnut.2025.1541734)

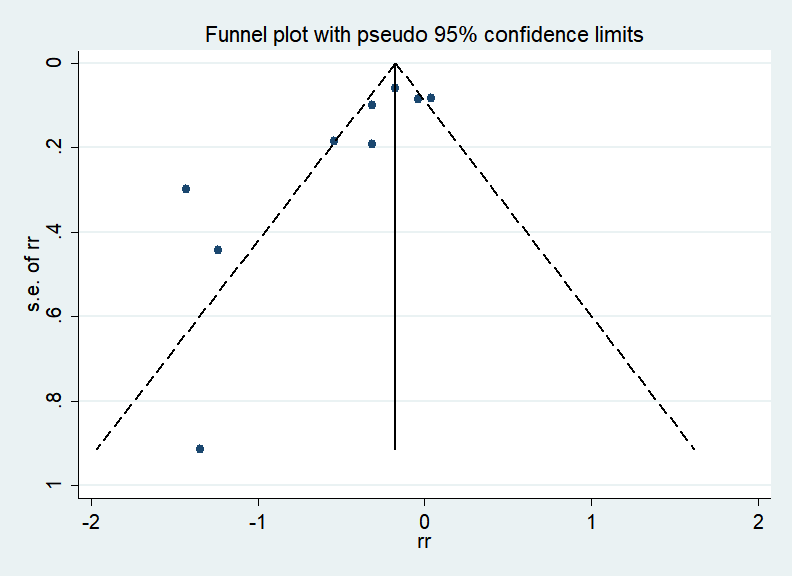

Supplement: Supplementary file 1 [file Image_1.tif]

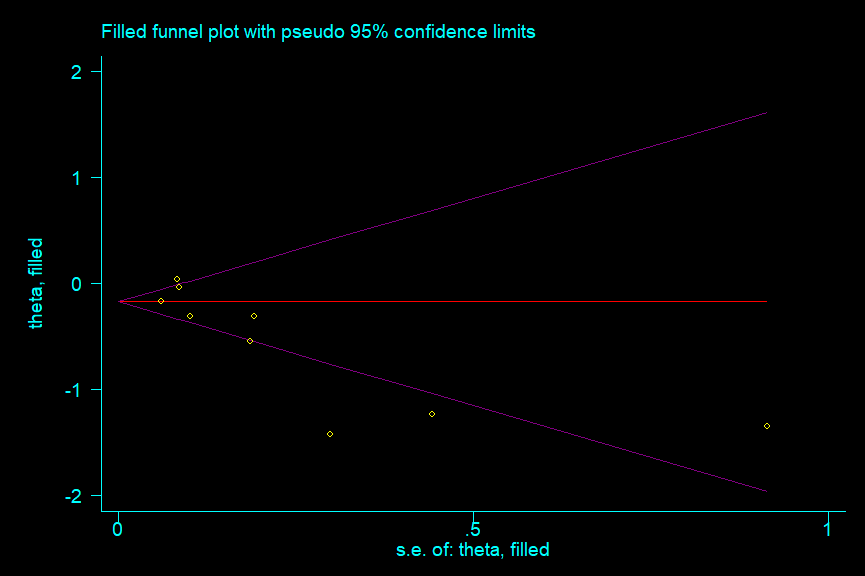

Supplement: Supplementary file 2 [file Image_2.tif]

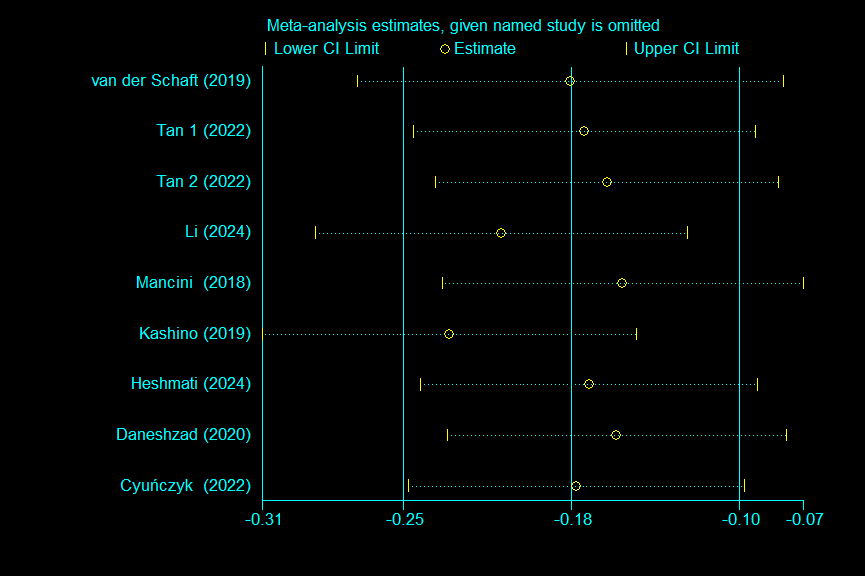

Supplement: Supplementary file 3 [file Image_3.tif]
