# Supplementary material for: Dietary total antioxidant capacity and risk of prediabetes and diabetes mellitus: a systematic review and dose-response meta-analysis of 170,919 participants
Source: Front Nutr. 2025 Feb 25;12:1541734. doi: 10.3389/fnut.2025.1541734 (PMC11893433; doi:10.3389/fnut.2025.1541734)
Supplement: Supplementary file 4 [file Table_1.DOC]

**Search terms in PubMed, EBSCO, Web of Science and Scopus**
((“dietary antioxidant capacity”[all fields] OR “dietary total antioxidant capacity” [all fields] OR “dietary TAC”[all fields] OR “non enzymatic antioxidant capacity”[all fields] OR “dietary antioxidant index”[all fields] OR “antioxidant capacity of diet”[all fields]) AND (“diabetes mellitus”[all fields] OR “diabetes”[all fields] OR “insulin resistance”[all fields] OR “hyperglycemia”[all fields] )

**Search terms in CNKI**

(膳食总抗氧化能力 OR 抗氧化剂) and 糖尿病

**Search terms in other sources**

The reference lists of all related articles and reviews were also manually searched.
